# Self-sustainable and recyclable ternary Au@Cu_2_O–Ag nanocomposites: application in ultrasensitive SERS detection and highly efficient photocatalysis of organic dyes under visible light

**DOI:** 10.1038/s41378-021-00250-5

**Published:** 2021-03-16

**Authors:** Tong Wu, Hui Zheng, Yichuan Kou, Xinyue Su, Naveen Reddy Kadasala, Ming Gao, Lei Chen, Donglai Han, Yang Liu, Jinghai Yang

**Affiliations:** 1 College of Physics, Jilin Normal University, Siping, 136000 China; 2 Department of Chemistry, Towson University, Towson, MD 21252 USA; 3 School of Materials Science and Engineering, Changchun University of Science and Technology, Changchun, 130022 China

**Keywords:** Nanoparticles, Nanoparticles

## Abstract

Ternary noble metal–semiconductor nanocomposites (NCs) with core–shell–satellite nanostructures have received widespread attention due to their outstanding performance in detecting pollutants through surface-enhanced Raman scattering (SERS) and photodegradation of organic pollutants. In this work, ternary Au@Cu_2_O–Ag NCs were designed and prepared by a galvanic replacement method. The effect of different amounts of Ag nanocrystals adsorbed on the surfaces of Au@Cu_2_O on the SERS activity was investigated based on the SERS detection of 4-mercaptobenzoic acid (4-MBA) reporter molecules. Based on electromagnetic field simulations and photoluminescence (PL) results, a possible SERS enhancement mechanism was proposed and discussed. Moreover, Au@Cu_2_O–Ag NCs served as SERS substrates, and highly sensitive SERS detection of malachite green (MG) with a detection limit as low as 10^−9^ M was achieved. In addition, Au@Cu_2_O–Ag NCs were recycled due to their superior self-cleaning ability and could catalyze the degradation of MG driven by visible light. This work demonstrates a wide range of possibilities for the integration of recyclable SERS detection and photodegradation of organic dyes and promotes the development of green testing techniques.

## Introduction

Surface-enhanced Raman scattering (SERS) is characterized by significant amplification of the Raman intensity of analytes adsorbed on rough nanoscale substrate surfaces^1^. As SERS can offer intrinsic fingerprint information about specific molecules, SERS based on molecular vibrations and/or rotations has been identified as a promising spectral analytical technology^2,3^. In addition to its ultrahigh detection accuracy and trace-level sensitivity, SERS has the advantages of rapidity, specificity, nondestruction of analytes, and in situ identification, resulting in a wide range of applications in the fields of biosensors, analytical chemistry, food safety, and environmental science^4,5^. Given the importance of SERS-active substrates for the enhancement of the Raman signal, a considerable number of studies have been conducted on the design and preparation of various noble metal nanomaterials as SERS substrates due to their unique surface plasmon resonance (SPR)^6^. In the past few years, two-dimensional (2D) SERS substrates have been widely favored due to their uniform plasma distribution. In our previous work, we constructed noble metal/semiconductor composite SERS substrates on 2D polystyrene (PS) nanosphere arrays by magnetron sputtering and achieved highly sensitive SERS detection of various environmental pollutants^7,8^. However, these nanostructured SERS substrates exhibited several drawbacks, such as a complex synthesis, high cost, and a need for large-scale expensive equipment^9^. To reduce costs and realize practical application of SERS substrates, much work has focused on the preparation of SERS substrates immobilized on modified magnetic supports. These magnetic SERS substrates could be separated from the liquid environment by an external magnetic field, and the analytes adsorbed on the SERS substrates could be removed by specific organic solvents, which enabled recycling and reuse of the SERS substrates^10^. For example, Li et al. applied Fe_3_O_4_–Ag Janus composites prepared by a two-step solvothermal method as SERS substrates with good recyclability and obtained Raman signals of crystal violet at a low concentration of 10^–13^ M^11^. We previously reported the synthesis of Fe_3_O_4_@Au nanocomposites as a reusable SERS substrate for the SERS detection of residual thiram on apple peels^12^. Unfortunately, the magnetic SERS substrates tend to aggregate during the synthesis process owing to the intrinsic strong magnetic interaction between the magnetic supports, which hinders the enhancement of Raman signals^13^. Huang et al. produced magnetic polyphosphazene–Ag by adding a polyphosphazene polymer shell between the magnetic Fe_3_O_4_ cores and the Ag nanoparticles, which effectively stabilized the magnetic cores and prevented their uncontrolled accumulation^14^. However, as analytes are usually firmly attached to the surfaces of SERS substrates after detection, it is difficult to completely remove the analytes by simple solvent cleaning; rather, multiple time-consuming cleaning steps are often required.

Recently, recyclable SERS substrates with ultraviolet (UV)-induced self-cleaning properties have received considerable attention. The self-cleaning performance of SERS substrates is usually dependent on the photocatalytic activity of wide-bandgap semiconductors. For example, the band gaps of ZnO and TiO_2_ are 3.37 and 3.2 eV, respectively^15,16^. The formation of reactive oxygen species (ROS; superoxide radical anions (O_2_^•−^) and hydroxyl radicals (OH^•^)) formed by ZnO and TiO_2_ under UV light irradiation can cause degradation of the analytes adsorbed on SERS substrates^17^. However, ZnO and TiO_2_ semiconductors can be excited only by UV light, which accounts for ~4% of the total solar spectrum^18^. To take full advantage of solar energy, it is significant to explore self-cleaning SERS substrates driven by visible light, as it accounts for ~42% of the solar spectrum^19,20^. Since the semiconductor Cu_2_O has a good response to visible light, it is considered a suitable alternative to ZnO and TiO_2_^21^. Interestingly, Lin et al. found that due to the effect of vacancy defects in promoting the charge-transfer (CT) process and electrostatic adsorption, a single Cu_2_O superstructure particle could act as a SERS substrate and detect target molecules at a low concentration of 10^−9^ mol/L^22^. However, compared with those of ZnO and TiO_2_, the electrons (e^−^) and holes (h^+^) of Cu_2_O generated by visible light excitation are easily recombined, as Cu_2_O has a relatively narrow bandgap of 2.17 eV, which may decrease the photocatalytic efficiency^23^. Due to the strong SPR excitation of noble metals, modification of Cu_2_O with noble metal nanocrystals is an effective strategy to promote the photocatalysis of Cu_2_O. More importantly, combining noble metal nanocrystals with the semiconductor Cu_2_O not only dramatically boosts the self-cleaning ability but also enhances the SERS signals owing to the enhanced CT resonance and exciton resonance.

The formation of bimetallic Au/Ag nanostructure assemblies as SERS substrates is another broadly applicable strategy to improve the sensitivity of SERS detection. Among all noble metals, Ag nanocrystals have the best SERS activity, but Au nanocrystals are more stable than Ag nanocrystals. Combining Au and Ag can make full use of their individual advantages and synergistic effects for improved chemical and plasmonic properties^24^. Most recently, researchers found that sandwich structures prepared by embedding semiconductors in two layers of plasmonic noble metals showed very high Raman enhancement due to multidimensional plasma coupling^25^. Ternary nanocomposites can have more SERS-active hot spots at the interfaces between noble metals and semiconductors, and Raman signals can be enhanced by several times due to the combined effect of CT and electromagnetic enhancement^26^. In addition, ternary nanocomposites can greatly promote the photocatalytic process of the semiconductor in the middle layer. After combination with semiconductors, noble metals can trap electrons to accelerate the separation of photogenerated electron–hole pairs in semiconductors. The SPR of noble metals can significantly enhance the local electric field, which can increase the light-harvesting efficiency of semiconductors^27^. Furthermore, a Schottky barrier forms at the interfaces between semiconductors and noble metals, which acts as an e^–^ sink and suppresses the recombination of e^–^ and h^+^^28^.

**Scheme 1:**
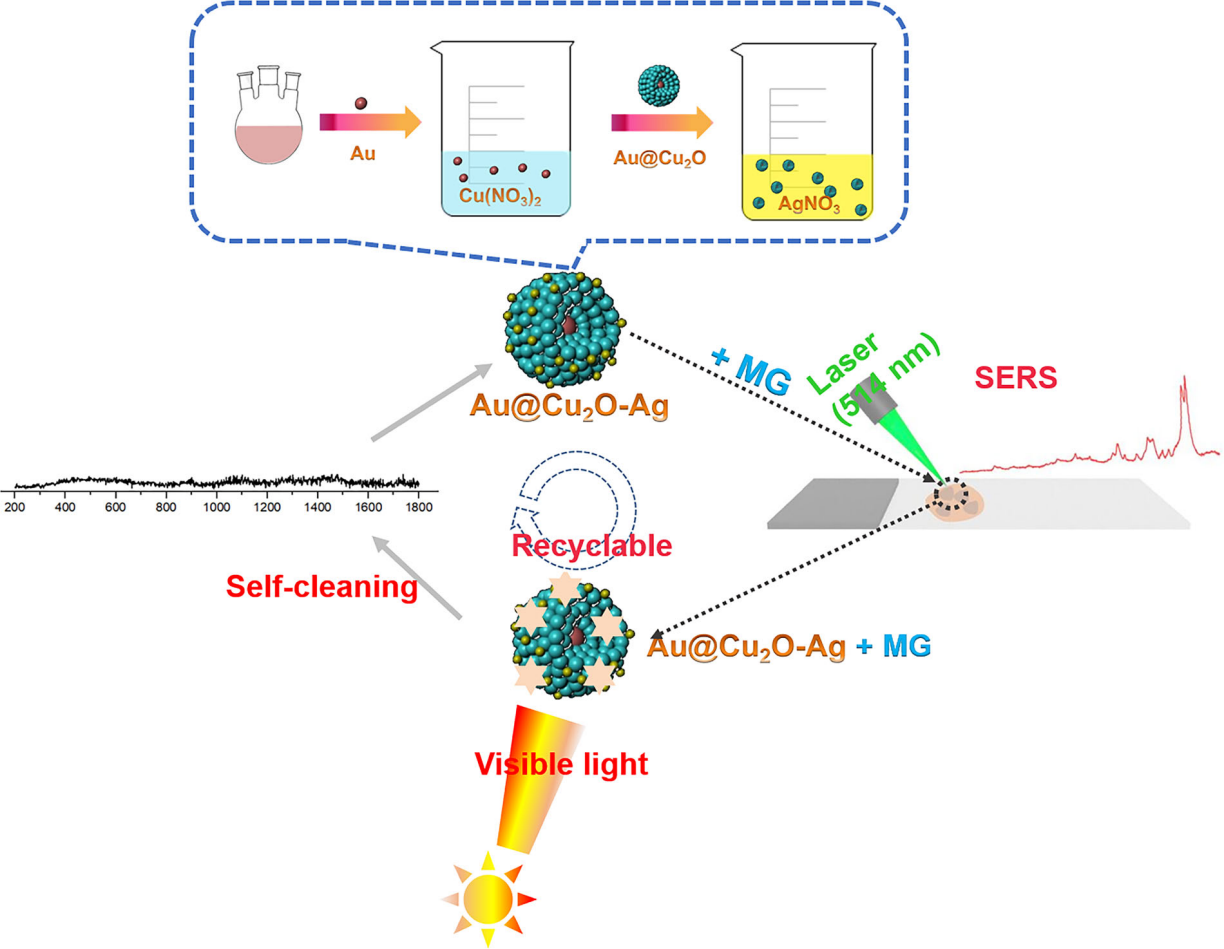
Preparation steps of Au@Cu2O–Ag NCs and their application as a self-cleaning SERS substrate for MG detection. Step-by-step preparation of Au@Cu_2_O–Ag NCs, SERS detection of MG, and catalytic degradation process of MG.

Inspired by the above ideas, we designed and synthesized novel ternary Au@Cu_2_O–Ag nanocomposites (NCs), which acted as a self-cleaning SERS substrate driven by visible light and a highly effective photocatalyst. The preparation procedure of Au@Cu_2_O–Ag NCs and the SERS detection and catalytic degradation process of malachite green (MG) are presented in Scheme 1. Using 4-mercaptobenzoic acid (4-MBA) as a reporter molecule, the effect of the Ag nanocrystal content on the Au@Cu_2_O surface on the SERS activity was discussed in combination with a three-dimensional finite difference time domain (FDTD) simulation. In addition, the self-cleaning ability of the ternary Au@Cu_2_O–Ag NCs was investigated. Our study not only expands the research on the SERS enhancement mechanism of ternary nanocomposites with core–shell–satellite structures but also opens new avenues to achieve the integration of recyclable SERS detection and highly effective catalytic degradation of organic pollutants.

## Results and discussion

### Structural and morphological analysis

**Fig. 1:**
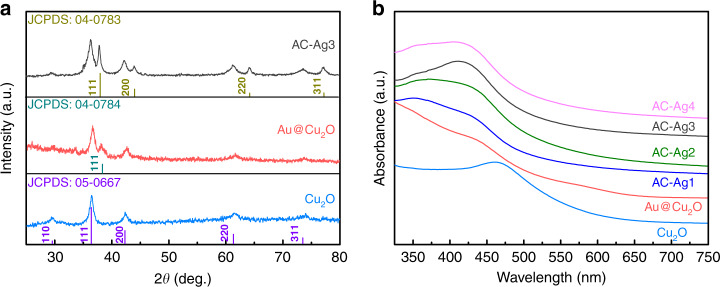
Structural and optical properties of the samples. **a** XRD patterns of the Cu_2_O nanocrystals, Au@Cu_2_O, and AC–Ag3 NCs; **b** UV–Vis absorption spectra of the Cu_2_O nanocrystals, Au@Cu_2_O, AC–Ag1, AC–Ag2, AC–Ag3, and AC–Ag4 NCs.

The XRD patterns of Cu_2_O, Au@Cu_2_O, and AC–Ag3 NCs are shown in Fig. 1a. The diffraction peaks of the Cu_2_O nanocrystals located at 2*θ* = 29.4, 36.5, 42.2, 61.5, and 73.7° were assigned to the characteristic crystalline planes of cubic Cu_2_O nanocrystals (JCPDS No. 05-0667). In addition to the diffraction peaks derived from the Cu_2_O nanocrystals, a weak diffraction peak attributed to the Au@Cu_2_O NCs was observed at 38.1° corresponding to the (111) crystal plane of Au (JCPDS No. 04-0784)^29^. The intensity of the diffraction peaks of Au is weak, probably because the Au core is wrapped in the Cu_2_O shell. The AC–Ag3 NCs exhibited broad diffraction peaks at 38.2, 44.4, 64.6, and 77.7°, which are characteristic of crystalline Ag (JCPDS No. 04-0783) and thus indicate the attachment of Ag nanocrystals to the Au@Cu_2_O NC surfaces^30^. Furthermore, the patterns of the Au@Cu_2_O NCs show all the main diffraction peaks of Ag, indicating the presence of Ag nanocrystals on the Au@Cu_2_O NC surfaces. Therefore, the XRD results preliminarily prove the successful synthesis of ternary Au@Cu_2_O–Ag NCs. The UV–Vis absorption spectroscopy of Cu_2_O nanocrystals shows a strong UV absorption at ~465 nm (Fig. 1b). Compared with the absorption peak of Cu_2_O nanocrystals, the absorption peak of Au@Cu_2_O NCs is blueshifted and broadened due to the interband transition and scattering of the Cu_2_O shells^31^. When Ag nanocrystals were immobilized on Au@Cu_2_O NC shells, the absorption bands shifted further to lower wavelengths due to the interaction between Au@Cu_2_O NCs and Ag nanocrystals^32^. The shift in the spectra of Au@Cu_2_O indicates that the Ag nanocrystals successfully adhered to the surfaces of the Au@Cu_2_O NCs.

**Fig. 2:**
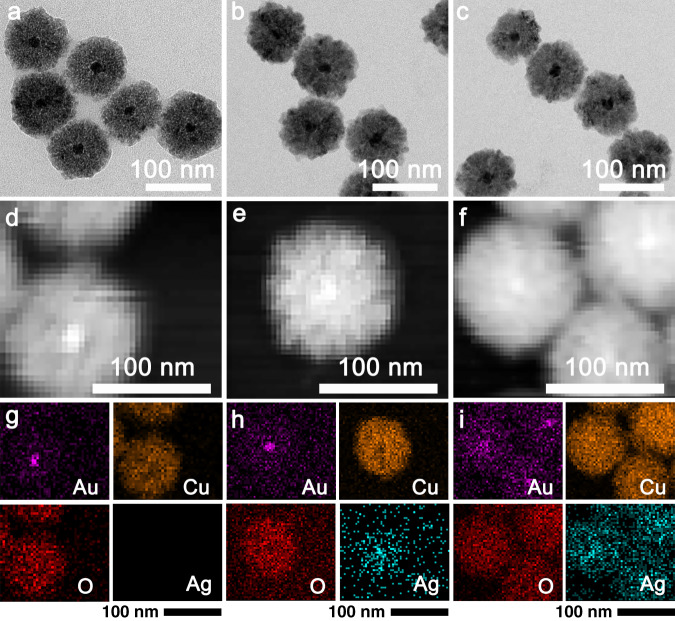
Morphology and element distribution of the samples. TEM images, HAADF–STEM images, and corresponding EDS results (Au, Cu, O, and Ag) of the Au@Cu_2_O (**a**, **d**, **g**), AC–Ag1 (**b**, **e**, **h**), and AC–Ag3 NCs (**c**, **f**, **i**).

The morphologies and sizes of the Au@Cu_2_O, AC–Ag1, and AC–Ag3 NCs were analyzed by TEM, as shown in Fig. 2a–c. The Au@Cu_2_O NCs exhibited a characteristic core–shell structure with Au in the center and Cu_2_O as the shell in the size range of 80–100 nm. The formation of the core–shell structure was facilitated by citrate ligands on the surface of the Au nanocrystals, which can bind Cu^2+^ ions and allow contact with Au nanocrystals during the reaction^33^. In contrast, no significant changes in the shape and size of the Au@Cu_2_O NCs were observed after the introduction of Ag nanocrystals, as shown in Fig. 2b, c. However, it is difficult to prove the presence of Ag nanocrystals on the Au@Cu_2_O NC surfaces based only on TEM images. The elemental distributions of the Au@Cu_2_O NCs, AC–Ag1 NCs, and AC–Ag3 NCs were determined from the high-angle annular dark-field scanning TEM (HAADF–STEM) images shown in Fig. 2d–f and the corresponding energy-dispersive spectroscopy (EDS) results shown in Fig. 2g–i revealing that Au was located at the center of the core–shell structure, while Cu and O were uniformly distributed on the Au core. Cu_2_O can act as a reducing agent to directly reduce Ag^+^ ions to form the ternary composite Au@Cu_2_O–Ag^34^. Ag nanocrystals were randomly and homogeneously immobilized on the shells of the Au@Cu_2_O NCs. In addition, the number of Ag nanocrystals on the Au@Cu_2_O NC shells increased substantially with increasing amounts of added Ag nanocrystals.

### SERS activity analysis

**Fig. 3:**
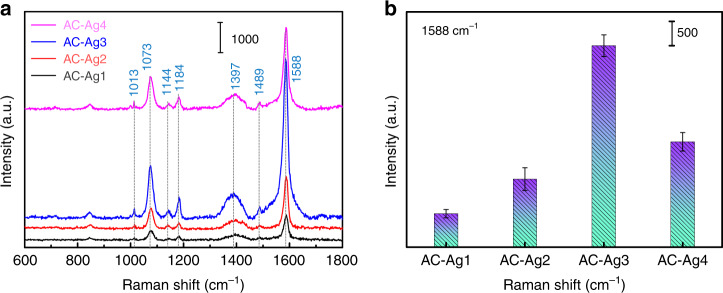
Detection of the reporter molecule 4-MBA. **a** SERS spectra and **b** SERS signal intensity at 1588 cm^−1^ of 4-MBA adsorbed on AC–Ag NCs. The error bars represent the standard deviations obtained from six independent measurements in (**b**).

To determine the optimal SERS substrate, AC–Ag NCs with different Ag contents were used as SERS substrates to detect 4-MBA in solutions of the same concentration, as shown in Fig. 3a. The detailed assignments of the bands in the SERS spectra of 4-MBA are listed in Table 1^35,36^. Figure 3b shows the SERS signal intensity of 4-MBA adsorbed on the AC–Ag(n) NCs at 1588 cm^−1^, and the error bars represent the standard deviations obtained from six independent measurements. Figure 3 shows that the SERS signal intensity of 4-MBA increased with increasing Ag content, and among all samples, the AC–Ag3 NCs gave rise to the highest intensity as a SERS substrate. Notably, the SERS signals of 4-MBA adsorbed on the AC–Ag4 NCs decreased unexpectedly. Given that physical and CT enhancement are two generally recognized mechanisms for SERS signal amplification, the above experimental phenomena are discussed based on these mechanisms.

**Table 1:** Assignments of bands in the SERS spectra of 4-MBA

Wavenumber (cm^−1^)	Band assignments*	Species
1013	In-plane ring breathing	b_2_
1073	In-plane ring breathing+ν(CS)	a_1_
1144	C–H deformation modes	b_2_
1184	C–H deformation modes	a_1_
1397	β(OH) + ν(C-ph)+in-plane ν(CC) + asymmetry ν(CO_2_)	b_2_
1489	ν (CC) + γ(CH)	
1588	Totally symmetric ν(CC)	a_1_

*ν* stretching, *β* bending. *For ring vibrations, the corresponding vibrational modes of benzene and the symmetric species under C_2v_ symmetry are indicated.

**Fig. 4:**
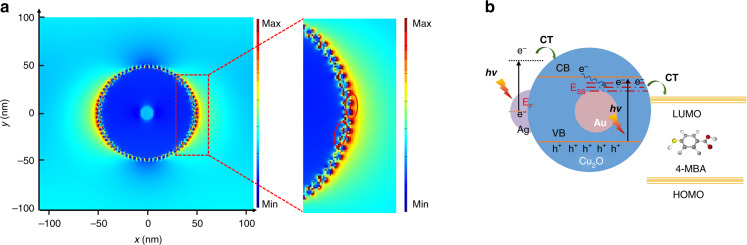
Analysis of the SERS mechanism. **a** Electric field distribution simulation by FDTD of AC–Ag3 NCs; **b** schematic of the charger transfer process between AC–Ag NCs and 4-MBA

The physical enhancement mechanism, also called the electromagnetic enhancement mechanism, is mainly based on the excitation of localized surface plasmon resonance (LSPR) on noble metal nanostructures^37^. In general, when the distance between adjacent noble metal nanocrystals is less than a certain value, a strongly localized and enhanced electromagnetic field is generated in the narrow interparticle gaps between the noble metal nanocrystals. The electromagnetic field intensity distribution of AC–Ag3 NCs was analyzed by FDTD simulation under periodic boundary conditions. As shown in Fig. 4a, the electric field enhancement was localized in the gap between the Ag nanocrystals, corresponding to the SERS-active sites commonly referred to as hot spots. In addition, a small number of hot spots were generated between the Cu_2_O shell and Ag nanocrystals. The SERS signal intensity is closely related to the number of hot spots in SERS substrates^38^. When the number of Ag nanocrystals on the Cu_2_O shell increased, the SERS signal intensity increased. Since the number of Ag nanocrystals on the surfaces of the AC–Ag3 NCs was higher than the number on the AC–Ag2 and AC–Ag1 NCs, it is reasonable that AC–Ag3 NCs displayed stronger SERS signals. In addition, the intensity of a SERS signal substrate is also relevant to the roughness of the SERS substrate. Excited photons generated by the incident light can collide with Ag nanocrystals several times on rough substrate surfaces during SERS detection, which can enhance the SERS signals. The unexpected decrease in the SERS signals of the AC–Ag4 NCs can be ascribed to the excess Ag nanocrystals, which easily led to the formation of Ag mass or even Ag shells and was unfavorable for the generation of hot spots. In contrast to chemical enhancement, electromagnetic enhancement is commonly thought to play a primary role as a contributor to SERS enhancement. However, the chemical contribution to SERS enhancement cannot be ignored.

In general, the SERS peaks of 4-MBA mainly consist of a_1_ symmetric vibration modes and b_2_ asymmetric vibration modes, and the enhancement of the b_2_ vibration modes is the result of the CT between the SERS substrates and 4-MBA, according to previous reports by Lombardi et al*.*^39^. The b_2_ vibration bands of 4-MBA adsorbed on the AC–Ag3 NCs at 1013, 1144, and 1397 cm^−1^ were enhanced compared with those of 4-MBA adsorbed on AC–Ag1 and AC–Ag2 NCs, verifying the increase in the CT process, which led to amplification of the SERS signals. The SERS signals of 4-MBA adsorbed on the AC–Ag4 NCs decreased unexpectedly, as the introduction of excessive amounts of Ag nanocrystals on the Au@Cu_2_O surfaces reduced the light absorption of Cu_2_O and the number of 4-MBA reporter molecules directly attached to the Cu_2_O surfaces. The Ag valence state in the AC–Ag3 NCs was analyzed by X-ray photoelectron spectroscopy (XPS), as shown in Supplementary Fig. S1. The peaks of AC–Ag3 NCs at 367.75 and 373.8 eV (splitting of 6.0 eV) between 3d_3/2_ and 3d_1/2_ were assigned to Ag 3d_5/2_ and Ag 3d_3/2_, respectively, indicating that Ag is present as Ag metal^40^. In comparison with that of pure Ag, the shift in the binding energy of Ag 3d provides evidence of an interaction between the Ag and Cu_2_O species^41^. To further elucidate the SERS chemical enhancement mechanism of 4-MBA adsorbed on the AC–Ag3 NCs, the SERS, and photoluminescence (PL) spectra were compared with those of the Cu_2_O nanocrystals and Au@Cu_2_O NCs, as shown in Supplementary Fig. S2. The SERS spectrum of the Cu_2_O nanocrystals exhibited no signals that could be attributed to 4-MBA. In contrast, the Au@Cu_2_O NCs exhibited weak SERS signals of 4-MBA, which could be ascribed to the promotion of CT between Cu_2_O and 4-MBA by the Au cores. In comparison, the AC–Ag3 NCs exhibited the strongest SERS signals, indicating that the Ag nanocrystals played a significant role in the SERS enhancement. Many studies have shown that a lower PL intensity indicates a higher carrier separation and transfer rate^42^. The PL peak intensity of the Au@Cu_2_O NCs was lower than that of the Cu_2_O nanocrystals because the Au cores acted as e^–^ traps to capture photogenerated e^–^ and increase the separation rate of photogenerated electron–hole pairs. When Ag was attached to the Au@Cu_2_O NC shells, the PL peak intensity further decreased, as modification with Ag resulted in the introduction of more e^–^ traps and further acceleration of e^–^ transfer. As shown in Fig. 4b, due to the LSPR effect of the noble metal Ag, the photoexcited electrons were injected into the conduction band (CB) of Cu_2_O and/or vibrationally relaxed to the surface-state energy level (*E*_SS_) of Cu_2_O to be transferred to the lowest unoccupied molecular orbital (LUMO) of 4-MBA in the ternary Au@Cu_2_O–Ag system. The CB and *E*_SS_ of Cu_2_O served as a bridge between Ag nanocrystals (donors) and probe molecules (acceptors)^43^.

**Fig. 5:**
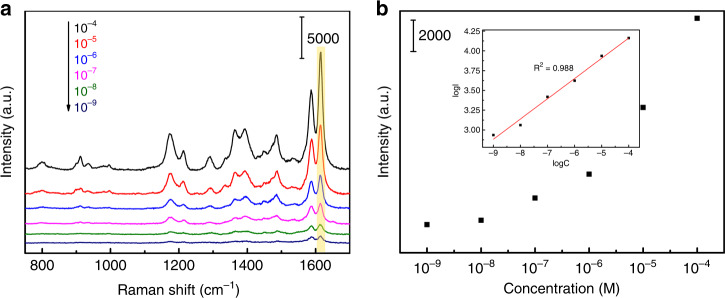
Sensitivity of the AC–Ag3 substrates. **a** SERS spectra of MG solutions with different concentrations adsorbed on AC–Ag3 substrates; **b** quantitative relation of the logarithm of the integrated SRES band at 1613 cm^−1^.

To evaluate the universality of the application of the ternary Au@Cu_2_O–Ag system as a SERS substrate, the SERS spectra of MG adsorbed on AC–Ag3 NCs were measured. Figure 5a shows the SERS spectra of MG solutions of different concentrations (10^−4^–10^−9^ M). The detection limit of the MG solution was as low as 10^−9^ M, indicating that the AC–Ag3 SERS substrates have a high SERS sensitivity. For comparison, the Raman spectra of solid MG powders are shown in Supplementary Fig. S3. The Raman shifts of solid MG powders and MG adsorbed on AC–Ag3 NCs and the corresponding detailed peak assignments are listed in Supplementary Table S1^44^. The relationship between the concentration of the MG solution and the SERS intensity at 1613 cm^−1^ is shown in Fig. 5, revealing that the decrease in the MG concentration is correlated with gradually weakened SERS signal intensities. The relationship between log C (logarithmic concentration) and log I (logarithmic intensity) shown in the inset of Fig. 5b is approximately linear, following the Langmuir isotherm model for the adsorption of target analytes^45^. In addition, the enhancement factor (EF) of the AC–Ag3 SERS substrates was assessed using the following formula^46^:

1 $${\it{{\mathrm{EF}}}} = \frac{{I_{{\it{{\mathrm{SERS}}}}}N_{{\it{{\mathrm{solid}}}}}}}{{I_{{\it{{\mathrm{solid}}}}}N_{{\it{{\mathrm{SERS}}}}}}}$$

where *I*_SERS_ and *I*_solid_ are the SERS intensities of the MG solution (1.0 × 10^−4^ M) after adsorption on the Au@Cu_2_O–Ag NCs and the Raman intensity of the solid MG powders on the glass substrates, respectively. *N*_SERS_ and *N*_Solid_ represent the corresponding numbers of MG molecules in the laser spot. The EF was estimated to be 7.19 × 10^5^, and the detailed EF calculation is presented in the Supplementary Information.

### Photocatalytic and regenerability analyses

**Fig. 6:**
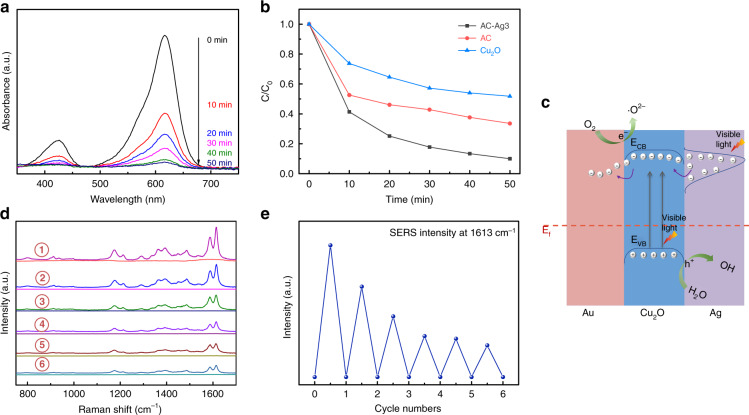
Photocatalytic activity, regenerability, and photocatalytic mechanism of the samples. **a** UV–Vis absorption spectra of MG solutions upon degradation by AC–Ag3 NCs. **b** Time-dependent photodegradation efficiency of MG in the presence of Cu_2_O nanocrystals, Au@Cu_2_O, and AC–Ag3 NCs. **c** Schematic illustration of the mechanisms of photodegradation driven by Au@Cu_2_O–Ag under visible-light irradiation. **d** SERS spectra of MG before and after the self-cleaning measurement carried out by the AC–Ag3 substrates and (**e**) the corresponding SERS intensities at 1613 cm^−1^ of the six recycling tests.

The self-cleaning ability is also an important indicator for evaluating the quality of SERS substrates. It can be quantified by investigating the photocatalytic performance of these substrates. Figure 6a shows the time-dependent absorption spectra of an MG solution in the presence of AC–Ag3 NCs under visible-light irradiation. Over time, the absorption intensity of MG decreased significantly, indicating the degradation of MG by the AC–Ag3 NCs. Figure 6b depicts the time-dependent concentration changes of MG induced by different samples relative to the initial concentration (C/C_0_). The rates of MG degradation by Cu_2_O and Au@Cu_2_O after visible-light irradiation for 50 min were only 48.2% and 66.4%, respectively. However, the degradation rate of MG over AC–Ag3 NCs reached 90.4% under the same conditions, which is 1.87 and 1.36 times higher than the rates of MG degradation by Cu_2_O and Au@Cu_2_O, respectively. These results imply that the designed novel ternary Au@Cu_2_O–Ag NCs have an optimal photocatalytic degradation ability for MG, which can be attributed to the synergistic effect among the three components. A possible mechanism for the photodegradation of MG catalyzed by AC–Ag NCs was proposed based on the above results, as presented in Fig. 6c. In general, the degradation of dye molecules proceeds mainly in two steps: light absorption by the dye molecules on semiconductor surfaces and photogeneration of free radicals (e.g., O_2_•^−^ and •OH)^47^. For the AC–Ag NCs, Cu_2_O produced photogenerated carriers (electron and hole pairs) under visible light irradiation. As Cu_2_O was sandwiched between Au and Ag and was in close contact with both metals, CT occurred at the interface between Cu_2_O–Ag and Cu_2_O–Au, effectively inhibiting carrier recombination. In contrast, for pure Cu_2_O nanocrystals, there were no prerequisites for CT between Cu_2_O and noble metals, leading to a low photodegradation efficiency of MG. Furthermore, considering that the work functions of Au (5.14 eV), Cu_2_O (5 eV), and Ag (4.26 eV) are different, a new Fermi-level equilibration was achieved as a result of CT^48^. In addition, compared with the Au@Cu_2_O NCs, two Schottky barriers exist at the interfaces of Cu_2_O–Ag and Cu_2_O–Au due to the differences in the Fermi levels (E_f_) of Au, Ag, and Cu_2_O, prolonging the lifetime of photoexcited carriers and thus further enhancing the photodegradation efficiency of MG^49^. It is noteworthy that Ag nanocrystals with LSPR properties can also boost the generation of carriers in Cu_2_O, which may be another cause of the increased degradation rate^50^. After a chain of reactions, e^–^ and h^+^ were trapped by surface-adsorbed O_2_ and H_2_O, respectively, to form O_2_^•−^ and •OH and eventually degraded MG. For these reasons, the ternary Au@Cu_2_O–Ag system displayed optimal photocatalytic performance.

In addition, the reusability of the prepared ternary Au@Cu_2_O–Ag SERS substrates was investigated. Figure 6d shows the SERS spectra of MG adsorbed on the AC–Ag3 NCs before and after self-cleaning under visible light irradiation. Accordingly, the SERS signals of MG were almost invisible after visible light irradiation, and the AC–Ag3 NCs maintained a high SERS performance even after six cycles. The SERS intensities at 1613 cm^−1^ of the six recycling tests are presented in Fig. 6e. Although the average SERS intensity decreased slightly owing to the reduction in the adsorption capacity, the requirements for the recyclable detection of MG were still satisfactory. Based on the above results, we can conclude that ternary Au@Cu_2_O–Ag NCs can be recycled in a process driven by visible light and have great potential as reusable SERS substrates to detect various organic pollutants.

## Conclusions

Ternary Au@Cu_2_O–Ag NCs with high SERS sensitivity were successfully synthesized. The XRD, UV–Vis, TEM, and EDS results confirmed the successful formation of Au@Cu_2_O–Ag NCs with core–shell-satellite nanostructures. The Au@Cu_2_O–Ag SERS substrates were optimized by adjusting the density of Ag nanocrystals on the Au@Cu_2_O nanocrystal surfaces. FDTD simulations indicated that the SERS signal intensity was closely related to the number of hot spots on the surfaces of the SERS substrates. The PL results showed that, compared with Au@Cu_2_O nanocrystals, Au@Cu_2_O–Ag NCs exhibited outstanding SERS enhancement, as the modification with Ag nanocrystals introduced more e^–^ traps and accelerated the e^–^ transfer between the SERS substrates and the probe molecules. Moreover, the Au@Cu_2_O–Ag NCs demonstrated excellent self-cleaning performance under visible light irradiation and could be recycled to maintain high SERS activity even after six cycles. This study not only expands studies on SERS enhancement and photocatalytic mechanisms but also opens the door to new possibilities in the integration of SERS detection devices and the photodegradation of organic dyes driven by visible light.

## Experimental section

### Synthesis of Au@Cu_2_O–Ag NCs

First, 100 mL of a tetrachloroauric(III) acid tetrahydrate (HAuCl_4_·4H_2_O) solution (2.4 × 10^−4^ M) and 4 mL of a trisodium citrate dihydrate (C_6_H_5_Na_3_O_7_·2H_2_O) solution (0.034 M) were mixed and heated for 30 min under reflux to obtain an Au colloid solution. Then, 0.5 g of polyvinylpyrrolidone (PVP K30) was added to 50 mL of a copper(II) nitrate trihydrate (Cu(NO_3_)_2_·3H_2_O) solution (0.005 M) and stirred for 6 min. After 1 mL of Au colloid solution was added to this mixture, 35 μL of a diluted hydrazine hydrate aqueous solution (N_2_H_4_·H_2_O, 85%) was added, followed by stirring for an additional 5 min. The mixed solution was centrifuged and rinsed five times with ethanol and deionized water to obtain Au@Cu_2_O NCs. Cu_2_O nanocrystals were prepared according to the same synthesis procedure as Au@Cu_2_O NCs but without the addition of the Au colloid solution. The obtained Au@Cu_2_O NCs were dispersed in 70 mL of deionized water. Then, 400 μL of silver nitrate (AgNO_3_) solution of different concentrations (2, 4, 6, or 8 mM) was added. After stirring for 30 min, Ag nanocrystals attached to the surface of the Au@Cu_2_O NCs to form Au@Cu_2_O–Ag NCs, which were denoted AC–Ag1 NCs, AC–Ag2 NCs, AC–Ag3 NCs, and AC–Ag4 NCs.

## Supplementary information

Supplementary Information
