# Supplementary material for: Self-sustainable and recyclable ternary Au@Cu2O–Ag nanocomposites: application in ultrasensitive SERS detection and highly efficient photocatalysis of organic dyes under visible light
Source: Microsyst Nanoeng. 2021 Mar 16;7:23. doi: 10.1038/s41378-021-00250-5 (PMC8433429; doi:10.1038/s41378-021-00250-5)
Supplement: Supplementary file 1 — Supplementary Information [file 41378_2021_250_MOESM1_ESM.docx]

Supporting Information for

Self-sustainable and recyclable ternary Au@Cu_2_O–Ag nanocomposites: application in ultrasensitive SERS detection and highly efficient photocatalysis of organic dyes under visible light

Tong Wu^1^, Hui Zheng^1^, Yichuan Kou^1^, Xinyue Su^1^, Naveen Reddy Kadasala^2^, Ming Gao^1^, Lei Chen^1^, Donglai Han^3^, Yang Liu^1,^* and Jinghai Yang^1,^*

^1^ College of Physics, Jilin Normal University, Siping 136000, China; wwwtttcn@126.com (T.W.); hzzh963@126.com (H.Z.); yichuankou@163.com (Y.C.K.); S15886017478@163.com (X.Y.S.); gaomingphy@126.com (M.G.); chenlei@jlnu.edu.cn (L.C.);

^2^ Department of Chemistry, Towson University, Towson, MD 21252, United States; naveenkadasala@gmail.com (N.R.K.);

^3^ School of Materials Science and Engineering, Changchun University of Science and Technology, Changchun 130022, China; dlhan_1015@cust.edu.cn (D.L.H.);

* Correspondence: liuyang@jlnu.edu.cn (Y.L.); jhyang1@jlnu.edu.cn (J.H.Y.)

The supporting information document consists of 8 pages, 3 figures, and 1 table.

**Materials**

Tetrachloroauric (III) acid tetrahydrate (HAuCl_4_·4H_2_O), trisodium citrate dehydrate (C_6_H_5_Na_3_O_7_·2H_2_O), copper (II) nitrate trihydrate (Cu(NO_3_)_2_·3H_2_O), polyvinylpyrrolidone (PVP K30), hydrazine hydrate aqueous solution (N_2_H_4_·H_2_O, 85%), silver nitrate (AgNO_3_) and malachite green (C_23_H_25_ClN_2_) were acquired from Sinopharm Chemical Reagent Co., Ltd. (Shanghai, China). 4-Mercaptobenzoic acid (4-MBA) was purchased from Sigma-Aldrich Chemical Co., Ltd. All the chemicals were used without further purification.

**SERS detection of MG**

1 mg of Cu_2_O nanocrystals, Au@Cu_2_O NCs and AC–Ag(n) NCs were added to 20 mL of 4-MBA solution, respectively. After centrifuging, washing and drying, SERS spectra were collected. MG solution was diluted with deionized water to obtain different concentrations (10^−4^ - 10^−9^ M) to determine the detection limit of Au@Cu_2_O. 1 mg AC–Ag3 NCs was immersed in 20 mL of MG solution with different concentrations under dark condition for 6 h, respectively. Subsequently, the MG-bound samples were centrifuged, washed with ethanol to remove unbound MG molecules and then dried for following SERS detection. The SERS spectra in this study were collected using a Renishaw inVia Raman system under 514 nm Ar^+^ ion laser excitation. In all Raman spectrum collection processes, the laser power is kept at 40 mW, the attenuation is 1%, and the accumulation time is 10 s. The Raman band of the silicon wafer was utilized to calibrate the spectrometer at 520.7 cm^−1^.

**Photodegradation process of MG**

20 mg of samples (AC–Ag3 NCs, Au@Cu_2_O NCs and Cu_2_O nanocrystals) was immersed in 20 mL of MG solution (10 mg/L) and left under dark condition for 30 min to reach adsorption equilibrium. Then, a CEL-S500 Xe lamp (Beijing Zhongjiao Jinyuan Technology Co., Ltd.) with a 420 nm cut-off filter was used as a visible light source to irradiate the above sample solution. The concentration of MG was monitored at regular intervals by using a UV–Vis spectrophotometer.

**Characterization**

Transmission electron microscopy (TEM) images were obtained through Hitachi H-800 transmission electron microscope (JEOL Ltd., Tokyo, Japan). X-ray powder diffraction (XRD) analysis was performed using a Rigaku D/MAX 3C X-ray diffractometer (Rigaku Corporation, Tokyo, Japan). The UV–Vis absorbance spectrum was recorded by a Shimadzu UV 3600 spectrophotometer (Shimadzu Corporation, Tokyo, Japan). Photoluminescence (PL) spectra were obtained at ambient temperature on a Renishaw in via micro-PL spectrometer with a He-Cd laser as the excitation light source at 323 nm. X-ray photoelectron spectra (XPS) analysis was performed using a Thermo Scientific ESACLAB 250 Xi A1440 system (Thermo Fisher Scientific, Waltham, MA, USA). All Raman spectra were detected by Renishaw inVia Raman spectrometer under a 514 nm Ar^+^ ion laser.

**Estimation of the enhancement factor**

The SERS enhancement factor (EF) was calculated as follows:

*EF*=$\frac{\text{I}_{\text{SERS}}\text{N}_{\text{Solid}}}{\text{I}_{\text{Solid}}\text{N}_{\text{SERS}}}\text{=}\frac{\text{I}_{\text{SERS}}}{\text{I}_{\text{Solid}}}\text{×}\frac{\rho{\text{×h×}\text{S}_{\text{laser}}\text{×N}}_{\text{A}}/M}{{\text{S}_{\text{laser}}}/{\text{S}_{\text{MG}}}}$ (1)

where *S_laser_* is laser spot size of 1 μm and the density (*ρ*) of the MG molecules is 1.131 g/cm^3^. The molar mass (*M*) is 111.16 g/mol and *h* is the effective layer depth of 19 μm. *N_A_* is the Avogadro constant. The band at 1613 cm^−1^ was selected to estimate *I* values. When determining *N_SERS_* in the illuminated volume of our Raman setup, it was assumed that MG molecules were absorbed as a monolayer on the surface of AC–Ag3 substrate. The surface area of a MG molecule (*S_MG_*) is ∼0.17 nm^2^. From the observed spectra, the ratio of intensities I_SERS_/I_Solid_ is 119.602, and the resulting EF is estimated to be around 7.19×10^5^.

**Figure S1**





**Fig. S1 The electronic states of Ag in AC–Ag3 NCs.** High resolution XPS scan of Ag 3d of AC–Ag3 NCs.

**Figure S2**


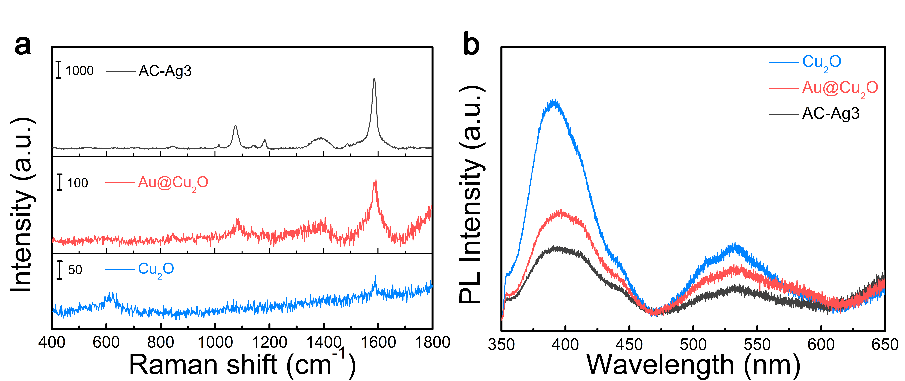


**Fig. S2** **Detection of reporter molecule 4-MBA and Photoluminescence (PL) spectra of samples.** SERS (a) and PL spectra (b) of 4-MBA adsorbed on Cu_2_O nanocrystals, Au@Cu_2_O and AC–Ag3 NCs.

**Figure S3**





**Fig. S3** **Raman spectra of target molecules.** Raman spectrum of solid MG powders.

**Table S1**. **Raman shifts of solid MG powders and MG absorbed on AC–Ag3 NCs and corresponding band assignments.**

| **Wavenumbers (cm^-1^)** | | **Band assignments*** |
| --- | --- | --- |
| Solid MG | AC–Ag3 |  |
| 803 | 799 | Ring C-H out-of-plane bending (γ(C-H)_ring_) |
| 915 | 911 | C-H out-of-plane bending, Ring skeletal vibration |
| 995 | 997 |  |
| 1175 | 1171 | In-plane vibrations of ring C-H (δ(C-H)_ring_) |
| 1217 | 1213 | C-H rocking (δ(C-H)_ring_) |
| 1293 | 1291 | ν(C-C)_ring_ |
| 1363 | 1365 |  |
| 1395 | 1390 | N-phenyl stretching, δ(C-H)_ring_ and (ν(C-C)_ring_) |
| 1484 | 1487 | ν(C-C)_ring_ and δ(CH_3_) |
| 1591 | 1588 | C-C stretching (ν(C-C)_ring_) |
| 1615 | 1613 | Ring C-C stretching (ν(C-C)_ring_) |
